# Electric‐Field Control of Propagating Spin Waves by Ferroelectric Domain‐Wall Motion in a Multiferroic Heterostructure

**DOI:** 10.1002/adma.202100646

**Published:** 2021-05-29

**Authors:** Huajun Qin, Rouven Dreyer, Georg Woltersdorf, Tomoyasu Taniyama, Sebastiaan van Dijken

**Affiliations:** ^1^ NanoSpin Department of Applied Physics Aalto University School of Science Aalto FI‐00076 Finland; ^2^ Institute of Physics Martin Luther University Halle‐Wittenberg 06120 Halle Germany; ^3^ Department of Physics Nagoya University Furo‐cho, Chikusa‐ku Nagoya 464‐8602 Japan

**Keywords:** domain‐wall motion, electic‐field control of magnetism, magnetoelectric coupling, multiferroic heterostructures, reconfigurable magnonics, spin waves

## Abstract

Magnetoelectric coupling in multiferroic heterostructures offers a promising platform for electric‐field control of magnonic devices based on low‐power spin‐wave transport. Here, electric‐field manipulation of the amplitude and phase of propagating spin waves in a ferromagnetic Fe film on top of a ferroelectric BaTiO_3_ substrate is demonstrated experimentally. Electric‐field effects in this composite material system are mediated by strain coupling between alternating ferroelectric stripe domains with in‐plane and perpendicular polarization and fully correlated magnetic anisotropy domains with differing spin‐wave transport properties. The propagation of spin waves across the strain‐induced magnetic anisotropy domains of the Fe film is directly imaged and it is shown how reversible electric‐field‐driven motion of ferroelectric domain walls and pinned anisotropy boundaries turns the spin‐wave signal on and off. Furthermore, linear electric‐field tuning of the spin‐wave phase by altering the width of strain‐coupled stripe domains is demonstrated. The results provide a new route toward energy‐efficient reconfigurable magnonics.

## Graphical abstract

Electric‐field control of propagating spin waves in an Fe/BaTiO_3_ multiferroic heterostructure is demonstrated experimentally. The underlying mechanism involves the concurrent motion of strain‐coupled ferroelectric domain walls and magnetic anisotropy boundaries and allows reversible tuning of the spin‐wave amplitude and phase. The results provide a new route toward energy‐efficient reconfigurable magnonics.

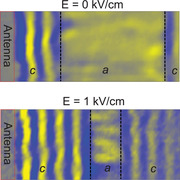

## 1 Introduction

Information encoding in the amplitude and phase of propagating spin waves enables a promising technology platform for ultrafast beyond‐CMOS computing without detrimental heating during device operation.^[^ ^1^, ^2^, ^3^, ^4^, ^5^ ^]^ Active control over spin waves in a magnetic material requires local variations of the effective magnetic field. Methods utilizing current‐driven Oersted fields,^[^ ^6^, ^7^, ^8^, ^9^ ^]^ electric fields and currents,^[^ ^10^, ^11^, ^12^ ^]^ and laser‐induced heating^[^ ^13^, ^14^, ^15^ ^]^ offer this essential functionality. Electric‐field manipulation of spin waves is particularly attractive because of its compatibility with on‐chip device integration and low‐power operation. Several physical mechanisms enable the coupling of electric fields to spin waves, including spin‐orbit interactions,^[^ ^16^, ^17^ ^]^ flexoelectricity,^[^ ^18^, ^19^ ^]^ and voltage‐controlled magnetic anisotropy (VCMA).^[^ ^20^ ^]^ VCMA, arising from changes in the occupancy of electronic orbitals near the interface of a magnetic film, has been exploited to excite ferromagnetic resonance (FMR),^[^ ^21^ ^]^ parametric magnetization oscillations,^[^ ^22^ ^]^ and propagating spin waves^[^ ^23^ ^]^ by radio‐frequency electric fields. Moreover, VCMA‐based reconfigurable magnonic crystals^[^ ^24^ ^]^ and spin‐wave nanochannels^[^ ^25^, ^26^ ^]^ have been proposed or demonstrated. As a pure interface effect, VCMA only enables electric‐field control of spin waves in ultrathin magnetic films, which limits the spin‐wave propagation length because of enhanced magnetic damping.

Magnetoelectric effects in multiferroic materials offer another path toward electric‐field control of spin waves. For instance, intrinsic coupling between electric and magnetic order parameters in single‐phase BiFeO_3_ has been shown to electrically alter the spin‐wave frequency.^[^ ^27^, ^28^ ^]^ Yet, weak magnetism at room temperature and strong magnetic damping complicate the emission and detection of spin waves in single‐phase multiferroics. Heterostructures comprising strain‐coupled ferroelectric and magnetic layers are a good alternative.^[^ ^29^, ^30^, ^31^, ^32^ ^]^ The large variety of available materials for magnetoelectric multiferroic composites offers flexible optimization of the coupling strength and spin‐wave transport properties. Moreover, as strain transfer to a magnetic film is not restricted to the interface region, thicknesses up to ≈150 nm can be used to limit magnetic damping.^[^ ^33^ ^]^ Thus far, electric‐field control of spin waves via strain coupling has been studied primarily in systems combining a magnetic layer and a piezoelectric substrate or film. For instance, electric‐field tuning of effective magnetic damping,^[^ ^34^ ^]^ FMR modes,^[^ ^35^, ^36^, ^37^, ^38^ ^]^ and surface modes,^[^ ^39^ ^]^ electric‐field excitation^[^ ^40^ ^]^ and manipulation^[^ ^41^ ^]^ of propagating spin waves, and reconfigurable spin‐wave routing^[^ ^42^ ^]^ have been demonstrated. In these examples, the application of an electric field across the piezoelectric layer alters the strength of magnetoelastic anisotropy within the electrode contact area of the magnetic film. Spin‐wave emission is attained when the resulting changes in effective magnetic field are driven by a radio‐frequency electric field, whereas shifts in the spin‐wave dispersion relation facilitate the manipulation of FMR and propagating modes.

Here, we report on a different strain coupling mechanism for deterministic electric‐field control over the amplitude and phase of propagating spin waves. Rather than relying on piezostrain, we strain‐couple an epitaxial ferromagnetic Fe film to a ferroelectric BaTiO_3_ substrate with regular polarization domains. The orientation of spontaneous polarization within the ferroelectric domains alternates between in‐plane and perpendicular. Because the two ferroelectric domains transfer different strains to the Fe film, a matching pattern of magnetic anisotropy domains with distinct spin‐wave transport properties forms in the ferromagnetic layer via inverse magnetostriction. Using broadband spin‐wave spectroscopy, super‐Nyquist sampling magneto‐optical Kerr effect (SNS‐MOKE) microscopy, and micromagnetic simulations, we demonstrate that concurrent electric‐field‐driven motion of ferroelectric domain walls in the BaTiO_3_ substrate and pinned magnetic anisotropy boundaries in the Fe film facilitates reversible switching of the spin‐wave amplitude and linear tuning of the spin‐wave phase.

## 2 Results

**Figure 1:**
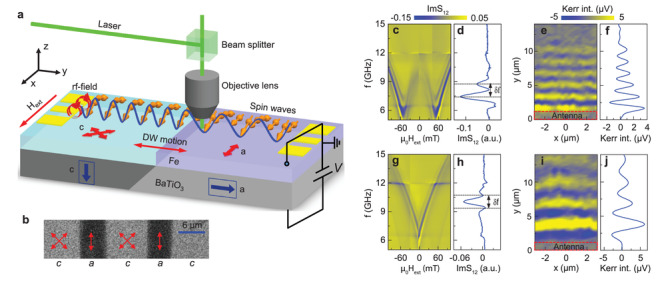
Spin‐wave transport in strain‐coupled domains of a Fe/BaTiO_3_ heterostructure. a) Schematic of the experimental configuration. The multiferroic heterostructure consists of a 26 nm‐thick ferromagnetic Fe film grown onto a ferroelectric BaTiO_3_(001) substrate with alternating in‐plane (*a*) and perpendicular (*c*) polarization domains. Strain transfer from the ferroelectric domains to the Fe film induces a regular modulation of uniaxial and biaxial magnetic anisotropy. Spin waves are excited by a microwave antenna and either inductively detected by a second antenna or imaged by SNS‐MOKE microscopy. An external magnetic bias field (*H*_ext_) is applied parallel to the ferroelectric domain walls. The ferroelecric domain walls that pin the anisotropy boundaries are moved by applying a perpendicular electric field across the BaTiO_3_ substrate. b) MOKE microscopy image of the Fe film recorded without bias field. The Fe film consist of alternating magnetic domains that fully correlate with the ferroelectric *a*–*c* domain structure of the BaTiO_3_ substrate. The double‐headed arrows indicate the symmetry and orientation of the strain‐induced magnetic anisotropy on top of the *a* and *c* domains. c,d) Spin‐wave transmission spectrum (ImS_12_) as a function of magnetic bias field and line profile measured at 70 mT for a single *c* domain. e,f) SNS‐MOKE microscopy image and line profile of a propagating spin wave on top of a *c* domain recorded at 14 GHz and 70 mT. g,h) Spin‐wave transmission spectrum (ImS_12_) as a function of magnetic bias field and line profile measured at 30 mT for a single *a* domain. The weak signal at low frequency in the contour plot is caused by the excitation of a FMR mode in a nearby *c* domain. i,j) SNS‐MOKE microscopy image and line profile of a propagating spin wave on top of an *a* domain recorded at 16 GHz and 70 mT.

**Figure** **1**a shows a schematic of the multiferroic heterostructure and measurement configuration. The ferroelectric BaTiO_3_ substrate consists of alternating domains with in‐plane and perpendicular polarization, as indicated by the blue arrows. Following conventions used in ferroelectrics, we label the domains as *a* and *c*, respectively. Because the crystal structure of BaTiO_3_ is tetragonal at room temperature, the *a* and *c* domains exhibit different structural symmetries within the substrate plane. For the (001) crystal orientation used in this study, the unit cells in the *a* domains are rectangular, whereas those of the *c* domains are square. Strain transfer from this ferroelectric domain pattern to a ferromagnetic film grown on top does therefore modulate the magnetic anisotropy. We selected Fe as the ferromagnetic material because it grows epitaxially onto BaTiO_3_(001), facilitating efficient strain transfer.^[^ ^43^ ^]^ Moreover, the small Gilbert damping parameter of Fe guarantees long‐distance spin‐wave propagation. We used molecular beam epitaxy (MBE) to grow an Fe film with a thickness of 26 nm. MOKE microscopy measurements performed after growth confirm full correlations between the stripe domains in the BaTiO_3_ substrate and the Fe film (Figure S1, Supporting Information). The strain‐induced magnetic anisotropy in the Fe film on top of the *a* and *c* domains is uniaxial and biaxial, respectively. In agreement with the negative magnetostriction constant of Fe, the easy anisotropy axis on top of the *a* domains aligns parallel to the ferroelectric domain walls, whereas the angle between the easy anisotropy axes on the *c* domains and the ferroelectric domain walls is 45° (see double‐headed red arrows in Figure 1a). The magnetic anisotropy boundaries in the Fe film are as narrow as the domain walls in the BaTiO_3_ substrate (≈2–5 nm).^[^ ^44^, ^45^, ^46^ ^]^ Figure 1b shows a MOKE microscopy image of the magnetic domain structure in zero magnetic field.

To facilitate spin‐wave characterization, we first covered the Fe film by an insulating TaO_*x*_ layer and then patterned 3 μm‐wide Au microwave antennas on top. Because the antennas are oriented parallel to the ferroelectric domain walls, the excited spin waves propagate perpendicular to the magnetic anisotropy boundaries. We studied spin‐wave transport by broadband spin‐wave spectroscopy and SNS‐MOKE microscopy. In the spectroscopy measurements, spin waves are excited by one microwave antenna and inductively detected by a second antenna using a vector network analyzer. The antennas are separated by 12 μm and spin‐wave transport over this distance is probed by recording the S_12_ scattering parameter. In the SNS‐MOKE microscopy setup, a microwave antenna is used for spin‐wave excitation and magneto‐optical images of propagating modes are obtained by scanning the sample in front of a focused laser beam. From the microscopy data, the spin‐wave amplitude, phase, and wavelength are derived. We performed the experiments with an external magnetic bias field (*H*_ext_) along the ferroelectric domain walls. A perpendicular electric field across the BaTiO_3_ substrate is used to move the ferroelectric domain walls.

We first discuss the propagation of spin waves in individual magnetic anisotropy domains. Figure 1c shows a contour plot of the imaginary part of the S_12_ scattering parameter as a function of magnetic bias field for a single *c* domain. As the bias field aligns along a hard axis of the biaxial anisotropy, the spin‐wave frequency decreases up to the magnetic anisotropy field μ_0_ *H*_ani,*c*_ = 48 mT before it increases at larger bias field. The frequency oscillations in the ImS_12_ signal signify spin‐wave transport between the two antennas.^[^ ^47^, ^48^ ^]^ From the line profile shown in Figure 1d, we derive a spin‐wave group velocity υ_g_ = δ*f* × *s* = 17 km s^−1^ at a bias field of 70 mT. In this expression for υ_g_, δ*f* is the frequency oscillation period (Figure 1d) and *s* is the spin‐wave propagation distance (12 μm). Fitting the field dependence of the FMR frequency for *H*_ext_ > *H*_ani,*c*_ to the Kittel formula results in a saturation magnetization *M*_s_ = 1705 kA m^−1^ (Figure S2a, Supporting Information), in good agreement with the Fe bulk value (*M*_s_ = 1710 kA m^−1^). The SNS‐MOKE microscopy image in Figure 1e shows the profile of a propagating spin wave in a magnetic *c* domain at 14 GHz. In this measurement, a bias field of 70 mT saturates the magnetization perpendicular to the spin‐wave wave vector, known as the Damon–Eshbach (DE) geometry. Averaging the SNS‐MOKE data along the *x* axis produces the line profile shown in Figure 1f. From the oscillating signal, we derive a spin‐wave wavelength λ = 1.74 μm and a decay length *l*_d_ = 4.5 μm (Figure S2b, Supporting Information). Figure 1g,h depicts spin‐wave transport data for a single *a* domain. Because the magnetic bias field aligns along the easy anisotropy axis of this domain, the spin‐wave frequency increases monotonically with the bias field. Fitting the field dependence of the FMR frequency to the Kittel formula using *M*_s_ = 1705 kA m^−1^, gives μ_0_ *H*_ani,*a*_ = 16.5 mT (Figure S3a, Supporting Information). From Figure 1h, we derive a spin‐wave group velocity of 17 km s^−1^ at 30 mT. Moreover, λ = 2.9 μm and *l*_d_ = 8.5 μm (Figure S3b, Supporting Information) at 16 GHz and 70 mT. The extracted parameters illustrate that strain‐coupling between the BaTiO_3_ substrate and the Fe film produces not only a lateral modulation of magnetic anisotropy, but also an alteration in the decay of propagating spin waves.

**Figure 2:**
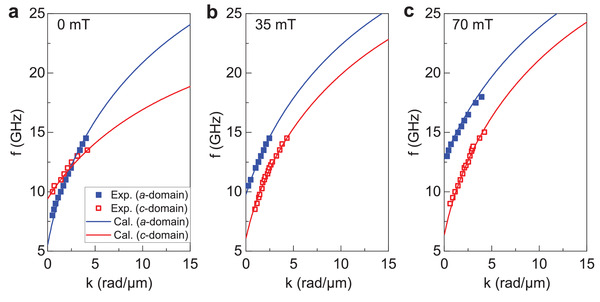
Spin‐wave dispersion relations. a–c) Measured (symbols) and calculated (lines) spin‐wave dispersion relations for the Fe film on top of ferroelectric *a* and *c* domains at a magnetic bias field of 0 mT (a), 35 mT (b), and 70 mT (c).

**Figure** **2** shows the spin‐wave dispersion relations for Fe on top of the ferroelectric *a* and *c* domains at three bias fields. The data depicted by symbols are extracted from SNS‐MOKE microscopy measurements and the lines are calculated using the Kalinikos and Slavin formula^[^ ^49^ ^]^ with experimentally derived values of *M*_s_ and *H*_ani_ as input parameters (see Experimental Section). In zero magnetic field, the spin waves propagate in the DE geometry in the magnetic *a* domains, whereas the magnetization and spin‐wave propagation directions make an angle of 45° in the *c* domains. Consequently, spin‐wave transport is more dispersive in the *a* domains. Together with a higher FMR frequency for the *c* domains (*H*_ani,*c*_ > *H*_ani,*a*_ at *k* = 0), this produces a crossing of the dispersion curves. With increasing bias field, the magnetization of the *c* domains coherently rotates toward the DE configuration. Using the Stoner–Wohlfarth model,^[^ ^50^ ^]^ we derive angles of 72° and 90° between the magnetization direction and the wave vector at 35 and 70 mT, respectively. Rotation of the magnetization direction increases the slope of the dispersion curve. Additionally, applying a magnetic bias field along the hard anisotropy axis of the *c* domains lowers the FMR frequency below the anistropy field (see also Figure 1c). Because the dispersion curve of the *a* domains shifts up with increasing bias field, the two dispersion relations no longer cross at 35 and 70 mT. At 70 mT, the magnetization in both anisotropy domains fully align along the magnetic bias field. In this uniform magnetization state without magnetic domain walls, the frequency separation between the two dispersion relations is about 5 GHz for small wave vectors. The distinct bias‐field dependencies of the dispersion relations for Fe on top of the ferroelectric *a* and *c* domains enables active programming of spin‐wave transmission signals across multiple stripe domains. We discuss this magnetic‐field effect next.

**Figure 3:**
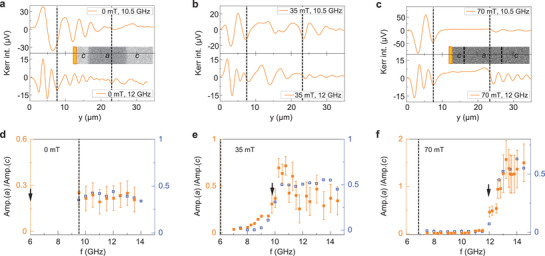
Magnetic‐field control of propagating spin waves. a–c) SNS‐MOKE microscopy measurements of spin‐wave transport across a *c*–*a*–*c* domain structure for three bias fields and two excitation frequencies. The insets in (a) and (c) show MOKE microscopy images of the sample configuration. The dashed lines in the line profiles mark the position of the *c*–*a* and *a*–*c* magnetic anisotropy boundaries. d–f) Ratio of the spin‐wave amplitude measured on the *a* domain at *y* = 12 μm and on the first *c* domain at *y* = 4 μm. The open squares depict results from micromagnetic simulations. The dashed lines and arrows mark the FMR frequency of the Fe film on top of the *c* and *a* domain, respectively. A spin‐wave signal is measured at *y* = 12 μm if the excitation frequency is larger than both FMR frequencies.

To illustrate reconfigurable spin‐wave transport, we measured the profile of propagating spin waves in a *c*–*a*–*c* domain structure using SNS‐MOKE microscopy. In the experiments, a microwave antenna excites spin waves in the first *c* domain. **Figure** **3**a–c shows results for three bias fields (0, 35, 70 mT) and two excitation frequencies (10.5, 12.0 GHz). At 0 and 35 mT, propagating modes are available in both anisotropy domains (Figure 2a,b) and, consequently, spin waves can efficiently propagate across the central *a* domain. Because of differing spin‐wave dispersion relations, the wavelength of spin waves converts at the *c*–*a* and *a*–*c* anisotropy boundaries (marked by dashed lines in Figure 3a,b). At 70 mT and 10.5 GHz, the absence of propagating modes in the *a* domain produces strong spin‐wave reflection at the *c*–*a* boundary (Figure 3c top panel). If the frequency is increased to 12.0 GHz, a FMR‐like mode in the *a* domain (*k* ≈ 0) enhances the transmission of spin waves into the second *c* domain (Figure 3c bottom panel). Figure 3d–f summarizes the dependence of spin‐wave transport on magnetic bias field and excitation frequency. In the graphs, we plot the ratio of the spin‐wave amplitude in the *a* domain at *y* = 12 μm and in the first *c* domain at *y* = 4 μm. With increasing bias field, the onset frequency of spin‐wave propagation across the *c*–*a* anisotropy boundary shifts up. The spin‐wave transmission signal can thus be turned on and off by a magnetic field at constant frequency. Micromagnetic simulations corroborate the experimental data (blue open squares in Figure 3d–f and Figure S4, Supporting Information).

**Figure 4:**
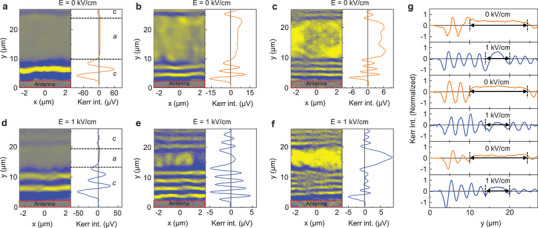
Control of spin‐wave transport by an electric field. a–f) SNS‐MOKE microscopy images and line profiles of propagating spin waves in an electric‐field controlled *c*–*a*–*c* domain structure. The upper and lower panels show results for *E* = 0 kV cm^−1^ and *E* = 1 kV cm^−1^. The spin‐wave frequency is 10.5 GHz (a,d), 12.0 GHz (b,e), and 13.5 GHz (c,f). g) Measurements of spin‐wave profiles recorded after repeated switching of the *c*–*a*–*c* domain structure. μ_0_ *H*_ext_ = 70 mT in all measurements.

We now demonstrate electric‐field control of spin‐wave transport in our strain‐coupled multiferroic heterostructure. In the experiments discussed here, a fixed magnetic bias field of 70 mT aligns the magnetization uniformly along the ferroelectric domain walls (data for μ_0_ *H*_ext_ = 0 and 35 mT are shown in Figure S5, Supporting Information). All‐electrical spin‐wave manipulation is attained by applying a perpendicular electric field across the BaTiO_3_ substrate. For fields pointing along the direction of perpendicular polarization, the *c* domains grow at the expense of the *a* domains through lateral motion of the ferroelectric domain walls.^[^ ^51^ ^]^ Because the magnetic anisotropy boundaries in the Fe film are pinned onto the ferroelectric domain walls in BaTiO_3_, they are forced to move along. The application of an electric field thus tunes the position of magnetic anisotropy boundaries and the size of magnetic anisotropy domains. **Figure** **4** shows SNS‐MOKE microscopy images and line profiles of propagating spin‐waves in an electric‐field controlled *c*–*a*–*c* domain structure. In zero electric field, the two *c* domains are separated by a 14 μm‐wide *a* domain (marked by dashed lines in Figure 4a–c). Applying an electric field of 1 kV cm^−1^ reduces the width of the central *a* domain to about 5.5 μm (Figure 4d– f). Because the dispersion relations of the *a* and *c* domains differ, the induced change of the magnetic anisotropy pattern alters the spin‐wave profile in the Fe film. Switching the electric‐field on and off produces a reversible modulation of spin‐wave transport, as demonstrated in Figure 4g. We note that the application of a voltage across the BaTiO_3_ substrate results in a metastable domain state. After the voltage is turned off, the magnetic anisotropy boundaries slowly move back to their initial position because of strain relaxation in BaTiO_3_. It takes about 12 h to retain the original domain state. Figure S6 in the Supporting Information provides more details on the electric‐field dependence of the domain width.

**Figure 5:**
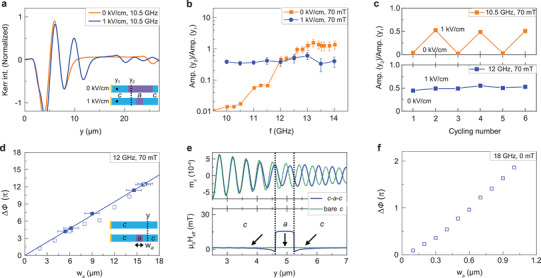
Electric‐field control of the spin‐wave amplitude and phase. a) SNS‐MOKE microscopy line scans of propagating spin waves in a *c*−*a*−*c* domain structure for *E* = 0 kV cm^−1^ and *E* = 1 kV cm^−1^. The schematics illustrate the size and position of the central *a* domain for the two electric fields. The frequency and magnetic bias field are 10.5 GHz and 70 mT. b) Ratio of the spin‐wave amplitude at *y*_2_ = 12 μm and *y*_1_ = 4 μm for both electric fields as a function of frequency. c) Variation of the spin‐wave amplitude at 10.5 and 12.0 GHz when the electric field is turned on and off repeatedly. d) Spin‐wave phase change Δϕ at *y* = 25 μm as a function of *a*‐domain width at 12.0 GHz and 70 mT. The solid and open symbols represent experimental and simulated data. The line corresponds to (*k*_*c*_ − *k*_*a*_) × *w*_*a*_, with the wave vectors taken from the dispersion relations shown in Figure 2c. e) Spin‐wave profiles for a *c*–*a*–*c* domain structure and a single *c* domain (top panel) and the effective magnetic field for both configurations (bottom panel). The data are simulated for 18 GHz and 0 mT. The domain walls have a 45° tail‐to‐tail/head‐to‐head structure. f) Spin‐wave phase change Δϕ as a function of *a*‐domain width.

The application of an electric field across the BaTiO_3_ substrate tunes both the amplitude and phase of propagating spin waves in the Fe film. Strong amplitude changes are attained below the FMR frequency of the *a* domain, that is, when the *a* domain blocks spin‐wave transport. Under this condition, moving the *c*−*a* anisotropy boundary out of or into an area between a prospective spin‐wave emitter and detector would turn the signal on or off. We illustrate this effect in **Figure** **5**a for a frequency of 10.5 GHz. The two depicted spin‐wave profiles correspond to the sample configuration under an electric field of 0 and 1 kV cm^−1^. The broad *a* domain in zero field suppresses the spin‐wave amplitude over a length of 14 μm, whereas the reduced *a* domain at 1 kV cm^−1^ does the same over a length of only 5.5 μm. Consequently, the spin‐wave amplitude in the Fe film jumps up in the area whose anisotropy changes from *a* to *c* by electric‐field‐driven motion of the *c*−*a* boundary. To quantify this effect, we plot the ratio of the spin‐wave amplitude at *y*_2_ = 12 μm and *y*_1_ = 4 μm (see schematic in Figure 5a). Figure 5b summarizes the frequency dependence of the amplitude ratio. Application of *E* = 1 kV cm^−1^ changes the spin‐wave amplitude most at low frequency and the effect diminishes toward the FMR frequency of the *a* domain (≈12 GHz). Above FMR, the spin waves propagate in both anisotropy domains. The larger spin‐wave amplitude at *y*_2_ for *E* = 0 kV cm^−1^ and *f* ≥ 12 GHz is explained by a slower decay of spin waves in the *a* domain, as derived previously from experiments shown in Figure 1. Electric‐field switching of the spin‐wave amplitude at 10.5 GHz is reversible (Figure 5c).

Active manipulation of the spin‐wave phase in our multiferroic heterostructure is based on electric‐field control over the size of magnetic anisotropy domains and wavelength conversions at magnetic anisotropy boundaries. At frequencies where spin waves do propagate across the alternating *c* and *a* domains, that is, *f* ≥ 12 GHz at a bias field of 70 mT, the spin‐wave wavelength converts up and down at the *c*–*a* and *a*–*c* boundaries, respectively (Figure 2c). Spin waves that propagate across the *c*–*a*–*c* domain structure therefore acquire a negative phase shift compared to spin waves that propagate over the same distance in a single *c* domain. Figure 5d quantifies this effect. In the graph, the absolute value of phase change Δϕ = |ϕ − ϕ_ref_| is plotted as a function of the *a* domain width (*w*_*a*_). Here, ϕ is the phase detected beyond the *a* domain at *y* = 25 μm and ϕ_ref_ is the reference phase at the same location for a single *c* domain (see schematic). Data extracted from experiments and simulations show a linear increase of Δϕ as the *a* domain widens. The phase change of the spin‐wave signal corresponds to (*k*_*c*_ − *k*_*a*_) × *w*_*a*_, as illustrated by the solid line in Figure 5d. At 12 GHz, the slope of the Δϕ − *w*_*a*_ curve corresponds to 2.45 rad μm^−1^. In other words, the externally applied electric field only needs to change the width of the *a* domain by 1.3 μm to induce a phase shift of π.

## 3 Discussion

Concepts for magnonic computing are based on active manipulation of the spin‐wave amplitude^[^ ^52^, ^53^ ^]^ or phase.^[^ ^54^, ^55^, ^56^, ^57^, ^58^, ^59^ ^]^ In previously studied magnonic systems utilizing VCMA or piezostrains, the application of an electric field alters the anisotropy strength in the gated area of a magnetic film. Propagation of spin waves along or across the gated area modifies both the spin‐wave amplitude and phase.^[^ ^25^, ^26^, ^41^ ^]^ The electric‐field control mechanism reported here is different, as it does not tune the strength of magnetic anisotropy in the Fe film on top of the individual *a* and *c* domains of the BaTiO_3_ substrate. Instead, the magnetic anisotropy boundaries in the multiferroic heterostructure follow the motion of ferroelectric domain walls under an applied electric field. This consorted motion offers nearly independent control over the amplitude and phase of propagating spin waves. Active programming of the spin‐wave amplitude is attained at frequencies where propagating modes are available in one of the two domain types. Under this condition, electric‐field‐induced motion of a magnetic anisotropy boundary away from or into the area between a spin‐wave source and detector reversibly alters the signal amplitude, an effect that could be exploited in low‐power spin‐wave filters. Continuous tuning of the spin‐wave phase relies on abrupt wavelength conversions between propagating modes in the *a* and *c* domains and electric‐field manipulation of the domain width. Control over the spin‐wave phase without suppression of the wave amplitude is particularly relevant for the realization of magnonic interference devices such as a Mach–Zehnder interferometer.^[^ ^54^, ^56^ ^]^ Besides the disentanglement of amplitude and phase manipulation, another attractive feature of our multiferroic heterostructure is the ability to control spin‐wave transport without magnetic bias field. Because efficient strain transfer from the ferroelectric *a* and *c* domains to the Fe film induces strong magnetic anisotropies, the magnetization aligns uniformly along one of the anisotropy axes in zero magnetic field (see MOKE microscopy image in Figure 1b). Spin waves can therefore propagate along the Fe film without disruption from magnetic disorder within the magnetic stripe domains.

To evaluate the scalability of phase control in zero magnetic field, we consider spin‐wave transport across a *c*–*a*–*c* domain structure with a 620 nm‐wide *a* domain at 18 GHz (Figure 5e). Because the spin‐wave wavelength converts up in the central *a* domain (see Figure 2a), the spin‐wave phase in the second *c* domain changes when an electric field alters the *a* domain width. The nearly linear dependence of the phase change Δϕ on *w*_*a*_ (Figure 5f) without accompanying suppression of the spin‐wave amplitude provides deterministic signal control for magnonic interference devices. For the short wavelength at 18 GHz, variation of the *a* domain width by about 600 nm already suffices to induce a phase shift of π. Electric‐field tuning of magnetic anisotropy domains thus allows for phase control on the nanoscale by utilizing high‐frequency spin waves in zero magnetic field (see Figure 2a). We note that the results depicted in Figure 5e,f are obtained with the magnetization aligning in a 45° tail‐to‐tail/head‐to‐head configuration (see arrows in Figure 5e). In this geometry, the effective magnetic field within the domain walls changes gradually and the spin waves transmit through the walls without amplitude loss. More narrow head‐to‐tail domain walls have been shown to filter spin‐wave signals by resonant reflection.^[^ ^60^ ^]^ We observe a similar effect in our multiferroic heterostructure when the remanent magnetization aligns into a 135° head‐to‐tail configuration. In this geometry, the transport of spin waves is more complex, as it depends on the *a* domain width and the excitation of domain‐wall resonances (Figure S7, Supporting Information).

## 4 Conclusion

We have demonstrated active spin‐wave manipulation by electric‐field controlled ferroelectric domain wall motion in a strain‐coupled multiferroic heterostructure. The new control mechanism provides independent tuning of the amplitude and phase of propagating spin waves, opening new avenues toward switchable and computational magnonics.

## 5 Experimental Section

### Sample Fabrication and Characterization

The Fe film was grown onto a BaTiO_3_(001) substrate using MBE. After etching the surface of the BaTiO_3_ substrate using Semico Clean 56 (Furuuchi Chemical Corp.) and rinsing with pure water, the substrate was transferred to a MBE system. The 26 nm‐thick Fe film was grown at a rate of ≈0.01 nm s^−1^ and a temperature of 300 °C. Deposition of Fe under these conditions resulted in a Fe[110] || BaTiO_3_[100]‐aligned epitaxial film with strain‐coupled domains.^[^ ^43^ ^]^ The domain structure of the Fe film using MOKE microscopy was imaged and the symmetry of magnetic anisotropy was determined by measuring the angular dependence of remanent magnetization (Figure S1, Supporting Information). For spin‐wave characterization, a 28 nm‐thick TaO_*x*_ insulating layer was sputtered on top of the sample and pairs of microwave antennas were patterned by direct laser‐writing lithography and lift‐off of a 3 nm Ta/120 nm Au layer. The antennas had a width of 3 μm and were separated by 12 μm. Perpendicular electric fields were applied across the BaTiO_3_ substrate by a bipolar power supply. The Fe film was used as grounded top electrode and double‐sided copper tape on the back of the BaTiO_3_ substrate as the bottom contact.

### Broadband Spin‐Wave Spectroscopy

The setup for all‐electrical spin‐wave characterization consisted of a two‐port vector network analyzer (Agilent N5222A) and a home‐built electromagnet probing station. The Gilbert damping parameter of the Fe film was determined by placing the sample face‐down onto a coplanar waveguide and measuring the FMR frequency and linewidth as a function of magnetic bias field (Figure S8, Supporting Information). Spin‐wave transmission between two parallel microwave antennas was probed by recording the S_12_ scattering parameter. To avoid nonlinear effects, the microwave excitation power was set to −10 dBm in all experiments. A frequency sweep method was used to record spin‐wave spectra at different magnetic bias fields. The measurement contrast was improved by subtracting a reference spectrum taken at 180 mT from the ImS_12_ data. An external magnetic bias field was applied along the ferroelectric domain walls.

### Super‐Nyquist Sampling Magneto‐Optical Kerr Effect Microscopy

A home‐built SNS‐MOKE microscope was used to image spin‐wave transport in the Fe film. In SNS‐MOKE, the laser frequency comb downconverted the excited GHz magnetization dynamics to an intermediate frequency ε, allowing tuning of the excitation signal to any frequency *f*_exc_ = *n* × *f*_rep_ + ε. At non‐zero ε and with the excitation synchronized to a laser repetition rate of 80 MHz, this downconversion occurs coherently, that is, the phase of spin waves relative to the excitation signal is preserved by lock‐in demodulation at ε.^[^ ^61^ ^]^ The SNS‐MOKE data were obtained by mounting the Fe/BaTiO_3_ sample onto a piezo‐controlled stage. Spin waves were excited by one of the microwave antennas and detected by a focused laser beam with a diameter of about 500 nm. Spatial mapping of the spin‐wave signal was performed by moving the sample stage along the spin‐wave propagation direction.

### Spin‐Wave Dispersion Relations

The spin‐wave dispersion relations for the Fe film on top of the ferroelectric *a* and *c* domains were calculated using the Kalinikos and Slavin formula:

(1) $$f=\frac{\gamma \;\mu_{0}}{2\pi }{\left[H_{\text{eff}}\left(H_{\text{eff}}+M_{\text{eff}}\left[1-F\sin^{2}\theta_{H}+\frac{M_{s}}{H_{\text{eff}}}F(1-F)\cos^{2}\theta_{H}\right]\;\right)\right]}^{1/2}$$

Here, $F=1-\frac{1-\text{exp}(-kd)}{kd}$ and θ_H_ is the angle between the wave vector and the magnetization direction. In the *a* domains, the magnetic bias field aligns along the easy anisotropy axis (θ_H_ = 90°). The effective magnetic field is therefore given by *H*_eff_ = *H*_ext_ + *H*_ani,*a*_, with μ_0_ *H*_ani,*a*_ = 16.5 mT. In the *c* domains, the magnetization rotated coherently with increasing bias field. The effective magnetic field corresponds to *H*_eff_ = *H_ext_cos(Φ) − H_ani,*c*_cos(4Φ)*,^[^ ^62^ ^]^ where Φ is the angle between the in‐plane magnetic bias field and the magnetization direction, and μ_0_ *H*_ani,*c*_ = 48 mT. Using the Stoner–Wohlfarth model,^[^ ^50^ ^]^ the angle *Φ* is estimated as 45° for 0 mT, 18° for 35 mT, and 0° for 70 mT. In the calculations, γ/2π = 29 GHz T^−1^ with a *g* factor of 2.09,^[^ ^63^ ^]^ *M*_s_ = 1705 kA m^−1^, and film thickness *d* = 26 nm were used for both the *a* and *c* domains.

### Micromagnetic Simulations

Micromagnetic simulations were performed using open‐source GPU‐accelerated MuMax3 software.^[^ ^64^ ^]^ The simulation area consisted of a 26 nm‐thick Fe film with a *c*−*a*−*c* domain structure. The area size was set to 65.5 μm along the *y*‐axis and 0.64 μm with 1D periodic boundary conditions along the *x*‐axis (mimicking infinitely long stripe domains). Spin‐wave reflections from the edges along *y* were suppressed by the addition of two 1 μm‐wide areas with strong magnetic damping. The simulation area was discretized into 4 × 4 × 13 nm^3^ cells. The *a* and *c* domains were defined by their uniaxial and biaxial magnetic anisotropy. The strengths of magnetic anisotropy were set to the experimentally derived values *K*_*a*_ = 15 × 10^3^ J m^−3^ and *K*_*c*_ = 44 × 10^3^ J m^−3^. The other input parameters were saturation magnetization *M*_s_ = 1705 kA m^−1^ and Gilbert damping parameter α = 0.003, both extracted from the experiments (Figures S2 and S7, Supporting Information), and exchange constant *A*_ex_ = 21 pJ m^−1^ from ref. [65]. Spin waves were excited locally by an in‐plane sinusoidal magnetic field with a strength of 1 mT. The excitation field was applied over a 400 nm‐wide area in the center of a *c* domain (marked as *y* = 0 in the graphs).

## Conflict of Interest

The authors declare no conflict of interest.

## Supporting information

Supporting Information

## Data Availability

The data that support the findings of this study are available from the corresponding author upon reasonable request.
