# Supplementary material for: Electric‐Field Control of Propagating Spin Waves by Ferroelectric Domain‐Wall Motion in a Multiferroic Heterostructure
Source: Adv Mater. 2021 May 29;33(27):2100646. doi: 10.1002/adma.202100646 (PMC11469113; doi:10.1002/adma.202100646)
Supplement: Supplementary file 1 — Supporting Information [file ADMA-33-2100646-s001.pdf]

© 2021 Wiley-VCH GmbH

ADVANCED MATERIALS

# Supporting Information

for *Adv. Mater.,* DOI: 10.1002/adma.202100646

# Electric-Field Control of Propagating Spin Waves by Ferroelectric Domain-Wall Motion in a Multiferroic Heterostructure

*Huajun Qin,* Rouven Dreyer, Georg Woltersdorf, Tomoyasu Taniyama, and Sebastiaan van Dijken**

# Supplementary Information for Electric-Field Control of Propagating Spin Waves by Ferroelectric Domain-Wall Motion in a Multiferroic Heterostructure

*Huajun Qin,* Rouven Dreyer, Georg Woltersdorf, Tomoyasu Taniyama, and Sebastiaan van Dijken**

Dr. Huajun Qin, Prof. Sebastiaan van Dijken
NanoSpin, Department of Applied Physics, Aalto University School of Science, FI-00076 Aalto, Finland
Email Address: huajun.qin@aalto.fi, sebastiaan.van.dijken@aalto.fi
Dr. Rouven Dreyer, Prof. Georg Woltersdorf
Institute of Physics, Martin Luther University Halle-Wittenberg, 06120 Halle, Germany
Prof. Tomoyasu Taniyama
Department of Physics, Nagoya University, Furo-cho, Chikusa-ku, Nagoya 464-8602, Japan

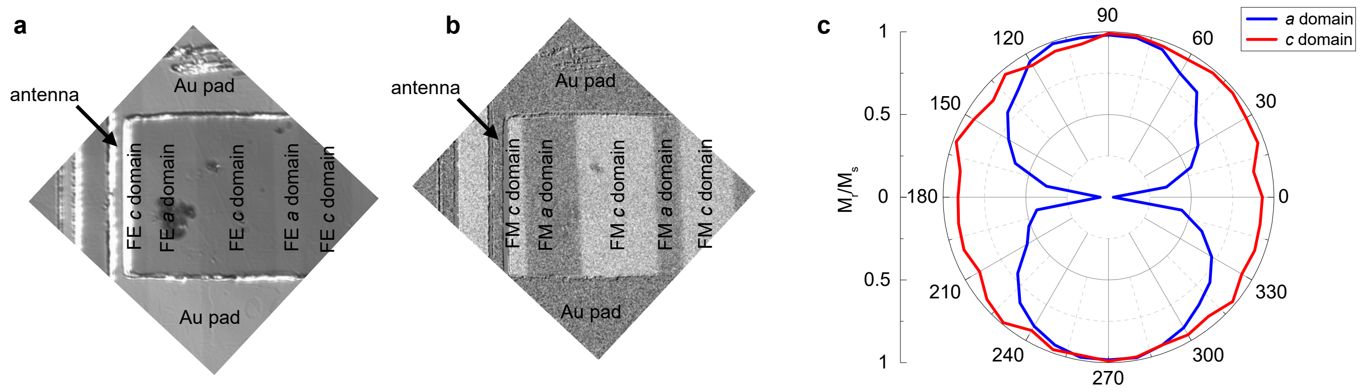


Figure S1. a,b) Polarization microscopy image (birefringence contrast in (a)) and MOKE microscopy image (magneto-optical contrast in (b) of the same Fe/$BaTiO_3$ sample location. Strain coupling between the ferroelectric (FE) $a$ and $c$ domains of the $BaTiO_3$ substrate and the Fe film produces an identical ferromagnetic (FM) domain structure. c) Polar plot of the normalized remanent magnetization on top of the $a$ and $c$ domains. Strain transfer from the $BaTiO_3$ substrate to the Fe film induces uniaxial and biaxial magnetic anisotropy in the $a$ and $c$ domains, respectively. The strength of magnetic anisotropy is derived from FMR measurements (see Figures S2 and S3).

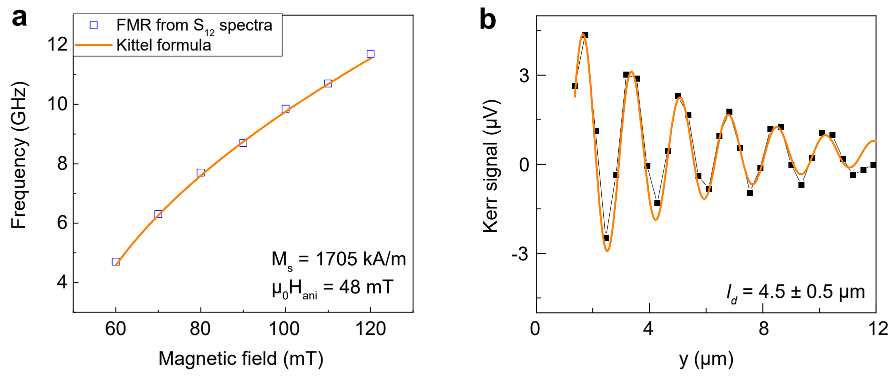


Figure S2. a) FMR frequency as a function of magnetic bias field recorded on top of a single $c$ domain. The line is a Kittel-formula fit to the experimental spin-wave spectroscopy data. From the fit and $\mu_0 H_{ani,c}$ = 48 mT (extracted from Figure 1c), we derive $M_s$ = 1705 kA/m. b) SNS-MOKE line profile of a propagating spin wave on top of a $c$ domain at 14 GHz and 70 mT. By fitting the data using $A\exp(-l_d y)\sin(By)$, we obtain a spin-wave decay length $l_d$ = 4.5 $\mu$m.

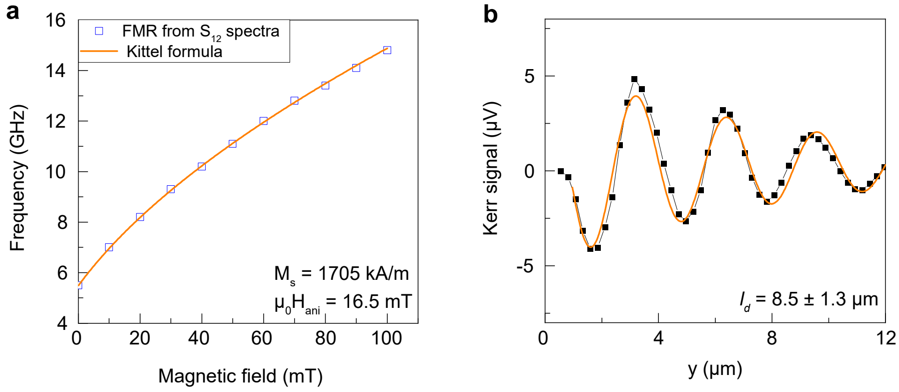


Figure S3. a) FMR frequency as a function of magnetic bias field recorded on top of a single $a$ domain. The line is a Kittel-formula fit to the experimental spin-wave spectroscopy data. From the fit and $M_s$ = 1705 kA/m (extracted from Figure S2a), we derive $\mu_0 H_{ani,a}$ = 16.5 mT. b) SNS-MOKE line profile of a propagating spin wave on top of an $a$ domain at 16 GHz and 70 mT. By fitting the data using $A\exp(-l_d y)\sin(By)$, we obtain a spin-wave decay length $l_d$ = 8.5 $\mu$m.

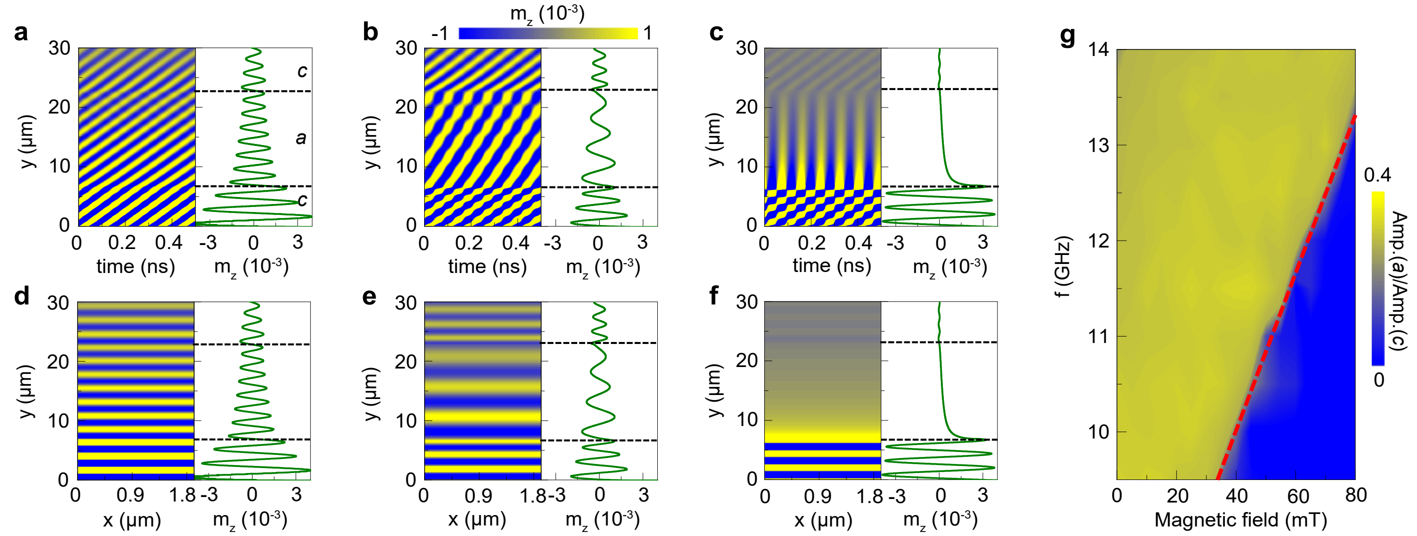


Figure S4. a-f) Micromagnetic simulations of spin-wave transmission through a $c - a - c$ domain structure at 12 GHz and a magnetic bias field of 0 mT (a, d), 35 mT (b, e), and 70 mT (c, f). Spin waves are excited in the first $c$ domain at $y = 0$. Panels (a-c) show the time evolution of spin-wave propagation under steady state excitation. Panels (d-f) depict the 2D spin-wave profiles. From the data, the amplitudes of propagating spin waves at $y = 12$ $\mu$m and and $y = 4$ $\mu$m are extracted. The ratio of these spin-wave amplitudes at different excitation frequencies are plotted in Figure 3d-f of the main manuscript. g) Contour plot of the amplitude ratio as a function of frequency and magnetic bias field. The red dashed line marks the field that is required for turning the spin-wave signal on and off.

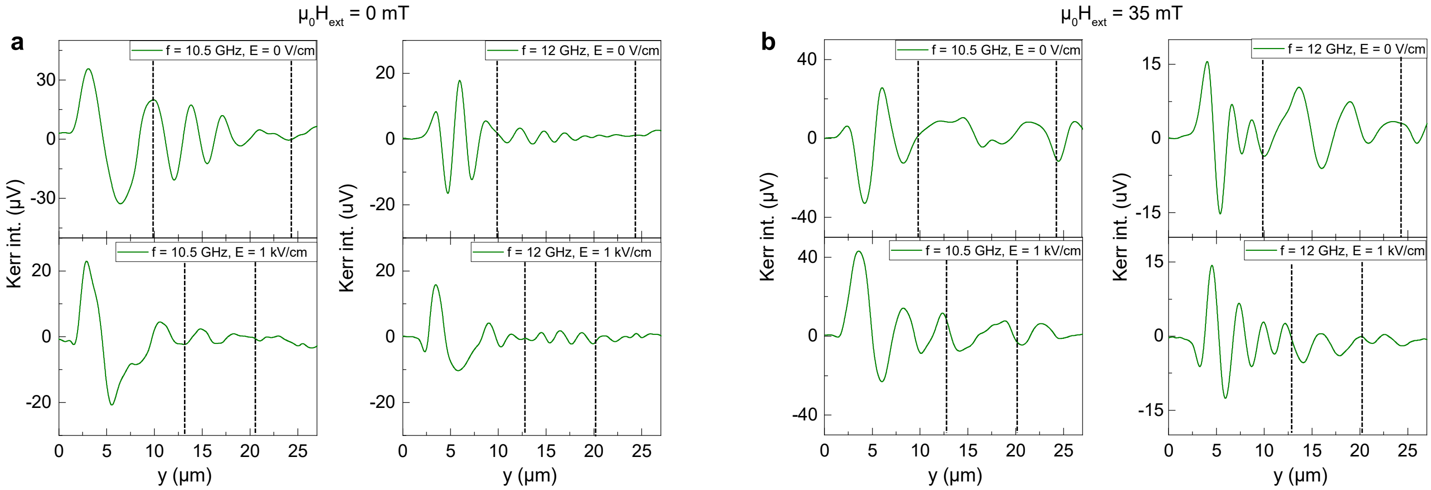


Figure S5. SNS-MOKE microscopy measurements of spin-wave transport across a $c - a - c$ domain structure for $\mu_0 H_{ext}$ = 0 mT (a) and $\mu_0 H_{ext}$ = 35 mT (b) and $E$ = 0 kV/cm (top panels) and $E$ = 1 kV/cm (bottom panels). The dashed lines mark the $c - a$ and $a - c$ magnetic anisotropy boundaries in the Fe film.

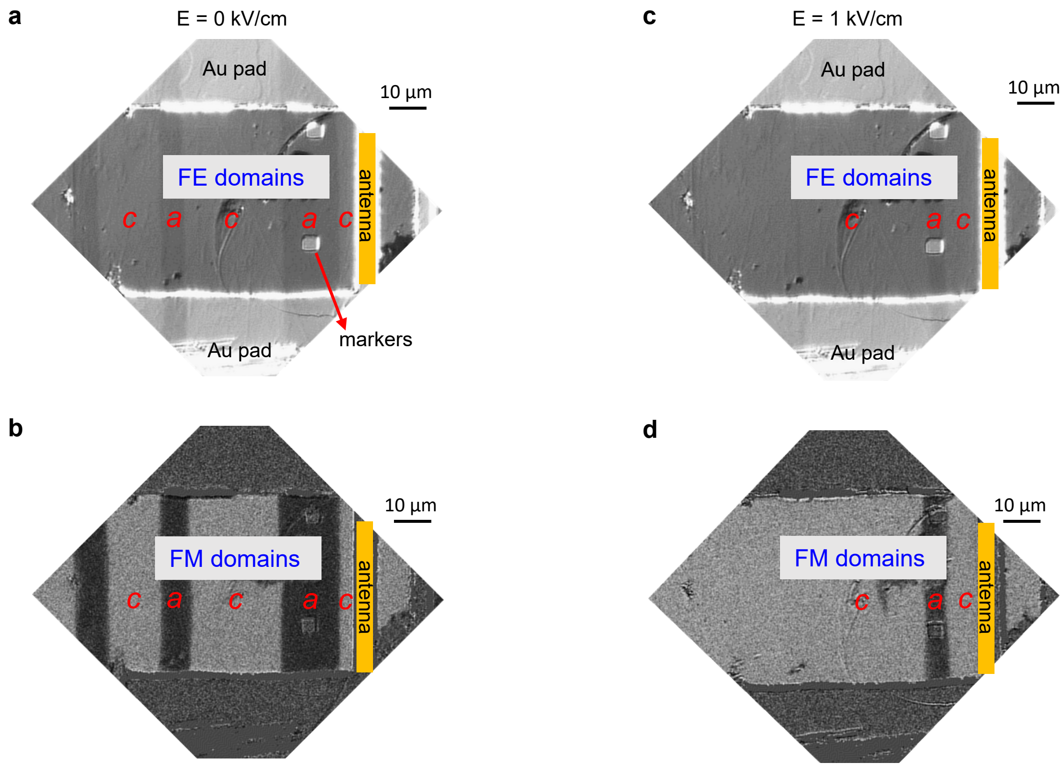


Figure S6. a-d) Polarization microscopy images showing ferroelectric contrast (a, c) and MOKE microscopy images depicting magnetic contrast (b, d) in the same sample area. Data are shown for $E$ = 0 kV/cm (a, b) and $E$ = 1 kV/cm (c, d). The images demonstrate that the ferroelectric (FE) and ferromagnetic (FM) stripe domains remain fully correlated during the application of an electric field. In the experiments, the perpendicular electric field aligns along the polarization direction of the $c$ domains. Consequently, the $c$ domains grow at the expense of the $a$ domains through lateral motion of the ferroelectric domain walls. The ferroelectric domain walls and pinned magnetic domain walls move in unison at $E$ = 1 kV/cm. In the experiments shown in Fig. 4 of the main manuscript, we applied an electric field of $E$ = 1 kV/cm to reduce the size of the $a$ domain from 14 $\mu$m to 5.5 $\mu$m. The SNS-MOKE microscopy measurements were performed immediately after turning the electric field off.

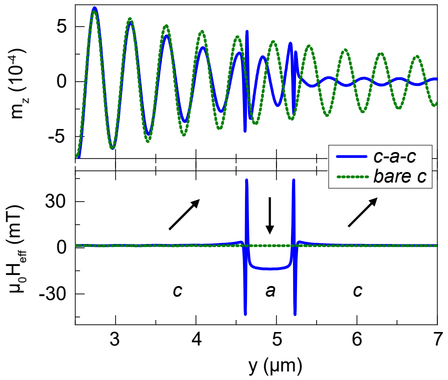


Figure S7. Spin-wave profiles for a $c-a-c$ domain structure and a single $c$ domain (top panel) and the effective magnetic field for both configurations (bottom panel). The data are simulated for 135° head-to-tail domain walls at 18 GHz and 0 mT. The non-uniform effective field in the narrow magnetic domain walls causes resonant spin-wave reflection. In combination with wavelength conversion in the $a$ domain, this produces a change in both the amplitude and phase of the transmitted spin-wave signal.

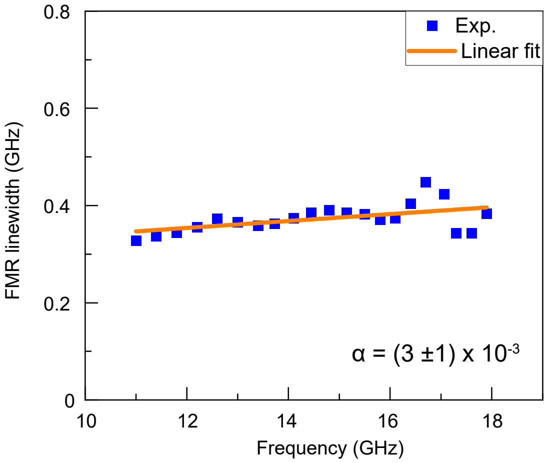


Figure S8. FMR linewidth as a function of frequency obtained by placing the sample face-down onto a coplanar waveguide. From a fit to the data using $\Delta f = 2\alpha f + v_g \Delta k$, we derive $\alpha = (3 \pm 1) \times 10^{-3}$ for the Fe film on top of $BaTiO_3$.
